# Supplementary material for: Active site specificity profiling datasets of matrix metalloproteinases (MMPs) 1, 2, 3, 7, 8, 9, 12, 13 and 14
Source: Data Brief. 2016 Feb 22;7:299–310. doi: 10.1016/j.dib.2016.02.036 (PMC4777984; doi:10.1016/j.dib.2016.02.036)
Supplement: Supplementary file 10 — Supplementary material [file mmc10.zip › WebPICS_hMMP9_T_1%/P3.html]

 

PICS results


|  |  |
| --- | --- |
| **P3\_A**  13 in 138 sites   9.4 %    effects > 10 perc. pnts.  (vice-versa in brackets)  P1\_N: 15.9 (20.6)   P1\_S: 13.0 (12.0)   P1prime\_M: 11.1 (23.9)   P2prime\_L: 19.9 (17.3) |  |
| **P3\_C**  2 in 138 sites   1.4 %    effects > 10 perc. pnts.  (vice-versa in brackets)  P2prime\_F: 45.7 (15.3) |  |
| **P3\_V**  13 in 138 sites   9.4 %    effects > 10 perc. pnts.  (vice-versa in brackets)  P1\_D: 16.6 (23.9)   P1prime\_M: 18.8 (40.6)   P2prime\_H: 15.9 (20.6) |  |
